# Delta corticomedullary apparent diffusion coefficient on MRI as a biomarker for prognosis in IgA nephropathy

**DOI:** 10.1080/0886022X.2024.2441394

**Published:** 2024-12-17

**Authors:** Zitao Wang, Ling Jiang, Fang Lu, Li Qian, Ying Pan, Chengning Zhang, Zhimin Huang, Ming Zeng, Bin Sun, Bo Zhang, Huijuan Mao, Yudong Zhang, Suyan Duan, Changying Xing, Yanggang Yuan

**Affiliations:** a Department of Nephrology, the First Affiliated Hospital of Nanjing Medical University, Nanjing Medical University, Nanjing, China; b Department of Radiology, The First Affiliated Hospital with Nanjing Medical University, Nanjing, Jiangsu Province, China

**Keywords:** IgA nephropathy, MRI, interstitial fibrosis, apparent diffusion coefficient, prognostic tool

## Abstract

**Objectives:**

To explore the association of the cortico-medullary difference in apparent diffusion coefficient (ΔADC) with clinicopathological parameters of disease activity at the time of biopsy, and with the prognositic risk stratification in IgA nephropathy (IgAN) patients.

**Methods:**

We included 112 patients with biopsy-proven IgAN who measured ΔADC. Patients underwent a kidney biopsy and diffusion-weighted magnetic resonance imaging within one week of the biopsy. Clinicopathological characteristics were compared according to different ΔADC levels. The effect of ΔADC on eGFR and kidney fibrosis was explored using multivariate regression and ROC analysis. An individual’s 5-year risk probability of progressing to ESKD or decreasing of eGFR > 50% was calculated by the guidelines-recommended international risk-prediction tool in IgAN. The effect of ΔADC on prognostic risk stratification was assessed. Net reclassification improvement (NRI) was used to evaluate the model performance.

**Results:**

The average ΔADC was 168.89 ± 85.1 x10^−6^ mm^2^/s. ΔADC levels decreased significantly with increasing chronic kidney disease (CKD) stages (*p* = 0.0038). Spearman correlation analysis revealed that ΔADC was positively correlated with eGFR, hemoglobin, serum albumin, while negatively correlated with levels of serum creatine (Scr), blood urea nitrogen (BUN), T score of Oxford classification and Lee grades (*p* < 0.05). Moreover, we showed that ΔADC was independently associated with eGFR (β = 0.04, 95% CI = [0.003, 0.077], *p* = 0.033) demonstrated by a backward stepwise multivariate linear regression analysis. Besides, ΔADC, a combination of ΔADC and eGFR showed an AUC of 0.776 (60% sensitivity and 85.3% specificity) and an AUC of 0.875 (100% sensitivity and 69.6% specificity) respectively for evaluating kidney interstitial fibrosis (IF) severity. Furthermore, ΔADC showed an AUC of 0.792 (95% CI 0.677-0.906) for differentiating higher progression risk categories from lower categories (specificity = 91.6%, sensitivity = 58.8%). The low-ΔADC group (≤ median value 167.1 × 10^−6^ mm^2^/s) was associated with 7.509-fold higher likelihood of higher progression risk compared to the high-ΔADC group (>167.1 × 10^−6^ mm^2^/s) in a fully-adjusted model. And reclassification analyses confirmed that the final adjusted model improved NRI.

**Conclusions:**

ΔADC was significantly associated with kidney function and enabled a reliable evaluation of kidney IF severity in IgAN patients. Low ΔADC can predict a high 5-year kidney progression risk in IgAN, independent of important clinical factors. Moreover, the predictive ability to identify patients at high risk of severe kidney fibrosis and adverse progression estimates with satisfactory accuracy, facilitating ΔADC a promising and noninvasive tool in complementarily evaluating disease activity and the prognostic risk stratification in patients with IgAN.

## Introduction

As a primary immune glomerulonephritis (GN), immunoglobulin A nephropathy (IgAN) is the leading cause of biopsy-reported GN worldwide [1]. The etiology of IgAN is complex and associated with genetic, infectious and environmental factors. The deposition of IgA in the glomerular mesangial area is the typical pathological feature of IgAN. Abnormal IgA molecules are often galactose - deficient, which facilitate the formation of immune complexes and the deposition in the kidneys, leading to the decline of renal function. The global incidence of IgAN is 2.5 per 100,000, and the prevalence varies in different countries, being highest in Asian countries, followed by European countries and the United States [2–4]. Moreover, patients with IgAN have increased mortality compared with matched controls, with one additional death per 310 person-years, reducing life expectancy by 6 years [5]. In addition to the high morbidity and mortality, the incidence rate is the highest in young adults. Approximately 30-40% of IgAN patients will develop end stage kidney disease (ESKD) within 20-30 years of diagnosis, resulting in a substantial public health burden [3, 6]. Given the high risk of progression and limited treatment options, precise risk stratification is essential for guiding treatment and resource allocation, which may be helpful for the guidance of making clinical decisions and reasonably allocating medical resources.

For decades, despite the highly variable clinical presentations and diverse histological pathologies that have been described in IgAN, substantial improvement has been made in the calculation of an individual’s 5-year risks of progressing to ESKD or decreasing of eGFR > 50% based on clinical and pathological characteristics at the time of kidney biopsy [7]. The 2021 KDIGO Clinical Practice Guideline recommends using the international IgAN prediction tool for accurately quantifying patients’ 5-year risk of kidney disease progression [8]. The guidelines recommend risk stratification for each patient so that high-risk patients may benefit from timely and individualized treatment [7, 8].

Advanced with high-resolution and noninvasiveness, multiparametric magnetic resonance imaging (MRI) has been revealed valuable for kidney diseases, especially in the early-stage implication of the functional alternation in various kidney diseases [9–11]. Accumulating data have pointed to the important role of quantitative diffusion weighted imaging (DWI) in clinical practice, which is a state-of-the-art imaging way based on the random Brownian motion or the free diffusion of water molecules in the tissues, consequently assessing tissue structure [12, 13]. Quantitated by the apparent diffusion coefficient (ADC), DWI has shown the ability of early diagnosis and staging of CKD as an accurate and noninvasive imaging technique [14]. ADC is the most widely-used clinical diffusion parameter generated by a monoexponential model, emerging as a complementary or alternative method to noninvasively evaluate kidney interstitial fibrosis (IF) [15, 16]. Moreover, growing evidence has demonstrated that, in contrast to separate cortical or renal ADC, the corticomedullary difference of ADC (ΔADC) on DWI exhibits a better correlation with interstitial fibrosis (IF) and tubular atrophy progression. Also, due to its ability to reduce inter-individual variability of the measured index, ΔADC on DWI is a reliable predictor of kidney function decline and dialysis initiation in patients with native kidney disease or kidney allografts [11, 17–19]. However, the association between ΔADC and clinicopathological parameters in IgAN patients remains largely unclear, and more importantly, whether it plays a prognostic role in risk stratification.

Therefore, this study was aimed to assess the association of ΔADC on DWI with clinicopathological parameters of disease activity at the time of biopsy, as well as the prognostic risk stratification in IgAN patients.

## Materials and methods

### Subjects

This cross-sectional study recruited 127 patients (aged 18-65 years) who were diagnosed with IgAN by renal biopsy in a single center between April 2020 and April 2022. The institutional review board approved the review by the ethics committee (2021 - SR − 569), and written informed consent was obtained from all enrolled patients. All of these patients underwent non - contrast - enhanced magnetic resonance imaging (MRI) of the kidneys, including T2-weighted imaging (T2WI), T1-weighted imaging (T1WI), and axial DWI, either prior to renal biopsy or within one week.

**Figure 1.**
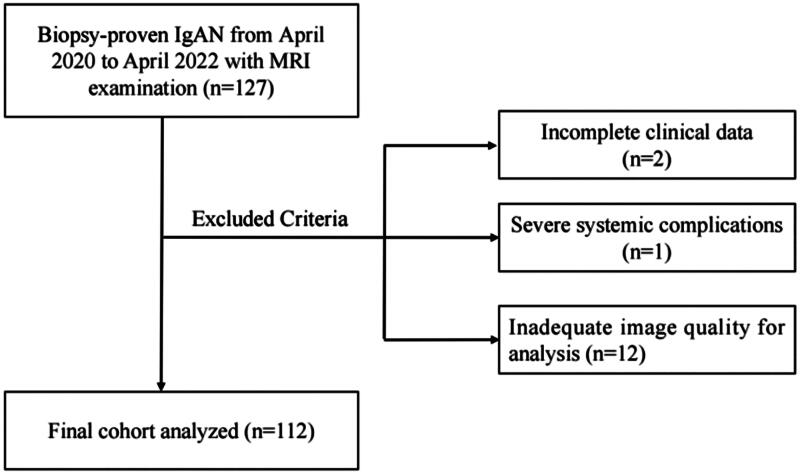
Flowchart of study participants. IgAN IgA nephropathy; MRI magnetic resonance imaging.

IgAN is diagnosed by renal biopsy, with IgA-predominant immunoglobulin deposition in the glomerular mesangial area as its pathological feature, according to the Kidney Disease: Improving Global Outcomes (KDIGO) 2021 Clinical Practice Guideline for the Management of Glomerular Diseases [8]. We excluded subjects with one of the following conditions: (a) with inadequate image quality for analysis (*n* = 12), including severe respiratory motion artifacts and unique physiological structure of kidney (such as polycystic kidney disease, medullary spongy kidney); (b) with severe systemic complications (*n* = 1); (c) with incomplete clinical data (*n* = 2), leaving a total of 112 patients for inclusion. The details of patient enrollment and exclusion were described in Figure 1.

### Clinical and laboratory parameters

The complete clinical and laboratory information at the time of renal biopsy of enrolled patients was collected, including age, gender, duration of kidney disease, body mass index, blood pressure, hypertension, urinary protein, estimated glomerular filtration rate (eGFR), blood urea nitrogen (BUN), serum creatinine (Scr), hemoglobin, 25-hydroxyvitamin D [25(OH)D], liver function tests, fasting blood glucose, triglyceride (TG), total cholesterol (TC), low-density lipoprotein cholesterol (LDL-C), high-density lipoprotein cholesterol (HDL-C), serum neutrophil gelatinase-associated lipocalin (NGAL), retinol binding protein (RBP), urine titratable acid, urine ammonia, urinary RBC, 24-h urinary electrolytes, serum immunoglobulin A (IgA), serum immunoglobulin G (IgG), complement C3, complement C4, etc. eGFR was estimated using Chronic Kidney Disease Epidemiology Collaboration (CKD-EPI) equation. In addition, the CKD stage was evaluated according to the K/DOQI guidelines.

### Kidney histopathology

Routine examination of every renal biopsy specimen was performed by light microscopy, electron microscopy, and immunofluorescence. All kidney biopsy slides were scored according to Lee’s pathological grade, Haas classification grade, Katafuchi semi-quantitative score, and the updated Oxford Classification (MEST-C) [20–23]. The Oxford MEST-C classification included five components and graded as follows: mesangial hypercellularity (M) as M0/M1 (M0: absent to ≤50% of glomeruli, M1: >50% of glomeruli with ≥ 4 mesangial cells per mesangial area); endocapillary cellularity (E) as E0/E1 (E0: absent, E1: present); segmental glomerulosclerosis (S) as S0/S1 (S0: absent, S1: present); tubular atrophy/interstitial fibrosis (T) as T0/T1/T2 (T0: absent to ≤25% of the cortex, T1: 25%-50% of the cortex, T2: >50% of the cortex); and cellular/fibrocellular crescents (C) as C0/C1/C2 (C0: absent, C1: 1%–24% of the glomeruli, C2: ≥25% of the glomeruli). The Oxford MEST-C classification was used to score Kidney IF, which was categorized into the mild group (T0), and moderate-to-severe (T1-2) [24–26]. Any scoring differences between the two pathologists were repeatedly reviewed until a consensus was obtained.

### International risk-prediction tool in IgAN

Individual’s 5-year risk probability of progressing to ESKD or decreasing of eGFR > 50% based on clinical and pathological characteristics including age, race, eGFR, SBP and DBP, proteinuria, use of ACEI/ARB and immunosuppression therapy, MEST-C score of Oxford classification at the time of kidney biopsy was calculated by QxMD (version 9.0, https://qxmd.com/calculate/calculator_499/international-igan-prediction-tool-at-biopsy-adults) [7]. The calculated formula is as follows:

$$P\text{​ }\text{redicted risk}(t)=1-S_{0}{\left(t\right)}^{e^{\text{LP}}}$$

$$\begin{array}{l} \text{LP}=-0.351(\sqrt{\text{eGFR}}-8.8)-0.0002(\text{MAP}-97)\\ \;-0.093[\text{ln}(\text{proteinuria})-0.09]+0.006[\text{MAP}\cdot \text{ln}(\text{proteinuria})-8.73]\\ \;+0.155M_{1}-0.131E_{1}+0.097S_{1}+0.607T_{1}+1.189T_{2}\\ \;+0.109T_{1}\cdot \text{ln}(\text{proteinuria})-0.339T_{2}\cdot \text{ln}(\text{proteinuria})\\ \;-0.016(\text{age}-38)+0.818\text{Chinese}\_\text{race}\\ \;+0.408\text{Japanese}\_\text{race}-0.431\text{Other}\_\text{race}\\ \;+0.246\text{RAA}S_{i}-0.225\mathrm{immunos}\text{ ​}\mathrm{uppression} \end{array}$$

Accordingly, the predicted progression risks were divided into two groups: at lower risk categories (predicted 5-year kidney progression risk ≤ 20%), and at higher risk categories (predicted 5-year kidney progression risk > 20%) [11].

### MRI acquisition

The MRI examinations were performed on a 3.0 T MRI scanner (MAGNETOM Skyra, Siemens Healthcare, Munich, Germany). Images for analysis of the kidney were comprised of T2WI, T1WI, and DWI in the axial pane. T1WI sequences were acquired with a repetition time (TR) of 75 ms, echo time (TE) of 1.22 ms, and flip angle of 70°. The voxel size was 1.3 × 1.3 × 5.9 mm, section thickness was 5 mm, field of view (FOV) was 339 mm and base resolution was 256 × 256, Time (min: sec) was 0:41. T2WI sequences were acquired with a TR of 4060 ms, TE of 87 ms and flip angle of 96°. The voxel size was 0.8 × 0.8 × 2.5 mm, section thickness was 2.5 mm, FOV was 240 mm and base resolution was 384 × 384, Time (min: sec) was 3:16. DWI had five diffusion gradient b values between 0 and 800 s/mm^2^ (0, 50, 200, 400, 800 s/mm^2^), with TR of 4100 ms, TE of 70 ms, voxel size of 1.8 × 1.8 × 5.0 mm, section thickness of 5 mm, time (min: sec) of 1:43, FOV of 240 mm and base resolution of 130. The analysis of the MRI images was blinded to clinicopathological parameters. Regions of interest (ROI) were determined on the T2WI (three ROI with an area of 30mm^2^ in cortex and medullae zones, respectively) as previously described and copied on the ADC map, produced by the Siemens MR system. All focal pathological areas (cysts, scars, and hematomas) were avoided in the ROI placement, aiming to cover a large and representative part of the cortex and medulla. ΔADC, the corticomedullary differences were used to reduce inter subject variability. ΔADC (cortical ADC - medullary ADC) was calculated to derive MRI signature that connects to clinicopathological parameters and prognostic risk stratification.

### Statistical analysis

Data were presented as mean ± SD, median and interquartile range or percentage. As appropriate, comparisons between groups were performed using one-way analysis of variance (ANOVA), Kruskal-Wallis test, or χ2 test. Spearman correlations were calculated to characterize the associations between ΔADC and baseline clinicopathological characteristics. Univariate and multivariate-linear regression analyses were performed to explore the independent factors that were attributed to eGFR and kidney IF in IgAN patients. The association of ΔADC and higher risk categories was determined by univariate and multivariate multinomial logistics regression analysis. Receiver operating characteristic (ROC) analyses with the area under the curve (AUC) were used to evaluate the diagnostic performance of MRI parameters for discriminating higher risk categories from lower categories, as well as differentiating moderate-to-severe from the mild IF group. We used net reclassification improvement (NRI) to assess how well the model predicted the outcomes. *P*-value < 0.05 was considered statistically significant. All statistics were done in IBM SPSS v.24.0 and R v.4.0.2.

## Results

### Characteristics of the study population

A total of 112 biopsy-proven IgAN patients were enrolled, with a mean age of 40.04 ± 13.69 years old and a median kidney disease duration of 8 (IQR 1, 24) months (Table 1). At the time of biopsy, the median urinary protein was 0.6 g/d (IQR 0.3, 1.42), and eGFR was 100 mL/min/1.73 m^2^ (IQR 75, 112.25). MRI was done within one week of the biopsy, and ΔADC was available in all enrolled patients with a level of 168.89 ± 85.1 × 10^−6^mm^2^/s. According to Lee’s grade, Lee grade III constituted 86.6% of the lesions, making it the most common pathological lesion type in enrolled patients, and 5 (4.5%) had Lee grade IV lesions. Meanwhile, there were 71 (63.4%), 6 (5.4%), 103 (92.0%), 10 (8.9%), and 13 (11.6%) patients with M1, E1, S1, T1-2, and C1-2, respectively. In addition, 66%, 30%, and 15% of enrolled IgAN patients had the predicted risk probability for ESKD or *a* > 50% reduction in eGFR within 5 years >5%, >10%, >20%, respectively.

**Table 1.** Baseline characteristics of the study population.

Parameter	Total (*n* = 112)	ΔADC >167.1 × 10-6 mm2/s (*n* = 56)	ΔADC≤ 167.1 × 10-6 mm2/s (*n* = 56)	P value
Age (years)	40.04 ± 13.69	40.32 ± 13.45	39.77 ± 14.04	0.832
Gender (male/female)	55/57	29/27	26/30	0.705
Duration of kidney disease (years)	8 (1, 24)	12 (1, 24)	4 (1.75, 24)	0.295
CKD stage (1/2/3a/3b/4)	72/25/8/5/2	39/13/3/1/0	33/12/5/4/2	0.371
**Clinical parameter**				
Body mass index (kg/m2)	20.8 (17.4, 25.42)	27.3 (23.4, 31.9)	26.1 (21.77, 31.9)	0.31
SBP (mmHg)	135.5 (121, 147)	130.5 (120, 144)	125 (115, 138.25)	0.141
DBP (mmHg)	91.5 (82, 102)	94 (84, 102)	85 (77, 97)	0.007
**Laboratory parameter**				
Urinary protein (g/d)	0.6 (0.3, 1.42)	0.55 (0.3, 1.12)	0.65 (0.3, 1.72)	0.337
eGFR (ml/min/1.73 m²)	100 (75, 112.25)	99 (83, 109.25)	102 (63.75, 115)	0.594
BUN (mmol/L)	5.08 (4.33, 6.32)	4.86 (4.39, 5.79)	5.3 (4.26, 7.28)	0.197
Scr (μmol/L)	77 (62, 96.25)	76 (57.75, 95.25)	78 (67, 97)	0.282
Hemoglobin (g/L)	132.96 ± 19.02	137.41 ± 19.17	127.86 ± 18.06	0.008
AST (u/L)	2.9 (2.42, 3.36)	19.05 (17.05, 23.33)	19.8 (17.6, 22.55)	0.584
ALT (u/L)	14.65 (11.07, 20.45)	17.3 (12, 25.82)	13.65 (10.57, 18.15)	0.065
Serum albumin (g/L)	38 (35, 41)	38 (36, 42)	37 (34, 39)	0.069
Fasting blood glucose (mmol/L)	4.48 ± 0.61	4.57 ± 0.63	4.38 ± 0.57	0.095
Triglyceride (mmol/L)	1.2 (0.93, 1.76)	1.23 (0.99, 1.78)	1.13 (0.92, 1.7)	0.4
Total cholesterol (mmol/L)	4.64 (3.98, 5.27)	4.56 (4.1, 5.27)	4.71 (3.9, 5.27)	0.859
LDL-C (mmol/L)	2.9 (2.42, 3.36)	2.94 (2.46, 3.22)	2.83 (2.37, 3.44)	0.859
HDL-C (mmol/L)	1.09 (0.95, 1.33)	1.04 (0.92, 1.23)	1.19 (0.96, 1.42)	0.077
Urine titratable acid	18 (12, 29)	18 (12.75, 28.25)	21 (12, 29.5)	0.744
Urine ammonia	35 (22.5, 54.5)	36 (22.5, 48)	32 (21.75, 61)	0.677
25(OH)D (nmol/L)	34 (25, 42)	35 (27.5, 40.25)	34.5 (25, 45.25)	0.747
Serum NAGL (ng/mL)	98 (54.5, 162.4)	68.7 (59.48, 109.52)	68.7 (58.67, 134.02)	0.396
IgG (g/L)	10.68 ± 2.72	10.76 ± 2.14	10.6 ± 3.22	0.757
IgA (g/L)	2.96 (2.38, 3.59)	2.74 (2.26, 3.62)	3 (2.5, 3.59)	0.524
IgM (g/L)	1.07 (0.8, 1.44)	1.04 (0.77, 1.32)	1.07 (0.82, 1.63)	0.3
C3 (g/L)	0.84 (0.76, 0.95)	0.84 (0.78, 0.93)	0.84 (0.74, 0.96)	0.602
C4 (g/L)	0.22 (0.19, 0.27)	0.22 (0.19, 0.26)	0.22 (0.18, 0.27)	0.974
RBP (mg/L)	41.75 (32.75, 52.25)	41.9 (33.12, 55.8)	40.7 (32.1, 48.95)	0.82
Urinary RBC (10 thousand/ml)	4.55 (1.65, 16.5)	4 (1.3, 17)	4.65 (2.08, 13.25)	0.539
24h Urinary Calcium	2.16 (1.52, 3.71)	2.92 (1.76, 3.99)	1.77 (1.02, 3.22)	0.004
24h Urinary Magnesium	3.1 (2.26, 3.88)	3.19 (2.33, 3.93)	2.95 (2.18, 3.73)	0.449
24h Urinary Phosphorus	15.65 (11.5, 20.4)	15.85 (11.78, 22.6)	15.85 (11.57, 18.97)	0.492
24h Urinary Sodium	113.65 (86.35, 158.45)	121.35 (95, 157.1)	108.1 (85.35, 172.22)	0.629
24h Urinary Chlorine	111.2 (84.57, 143.48)	115.85 (90.62, 143.48)	107.1 (76.6, 152.98)	0.551
24h Urinary Potassium	30.27 ± 11.26	29.42 ± 9.97	31.39 ± 12.12	0.349
Cortex ADC (× 10-6 mm2/s)	2099 (1999, 2190)	2113 (2007.25, 2196)	2081 (1982, 2188.75)	0.295
Medulla ADC (× 10-6 mm2/s)	1926 (1840, 2041)	1878.5 (1802.5, 1951)	1971.5 (1877, 2092.25)	0.005
ΔADC (× 10-6 mm2/s)	168.89 ± 85.1	233.55 ± 57.13	104.23 ± 53.27	< 0.001
**Pathological feature**		′		
Katafuchi score	40/12/30/22/8	19/8/15/12/2	21/4/15/10/6	0.483
(0/1/2/3/4)	10/97/5			
Lee’s grade (II/III/IV)	10/96/1/5	7/48/1	3/49/4	0.22
Haas classification(I/II/III/IV)		6/48/1/1	3/49/0/4	0.334
Oxford classification	41/71			
M (0/1)	106/6	24/32	17/39	0.239
E (0/1)	9/103	54/2	52/4	0.679
S (0/1)	102/8/2	5/51	4/52	1
T (0/1/2)	99/11/2	54/2/0	48/6/2	0.111
C (0/1/2)	40/12/30/22/8	51/4/1	48/7/1	0.748
**Predicted 5-year risk**				
>5%, n (%)	74 (66)	34 (61)	40(71)	0.318
>10%, n (%)	34 (30)	13 (23)	21 (38)	0.15
>20%, n (%)	17 (15)	4 (7)	13 (23)	0.035

CKD chronic kidney disease; BMI body mass index; SBP systolic blood pressure; DBP diastolic blood pressure; eGFR estimated glomerular filtration rate; BUN blood urea nitrogen; Scr serum creatine; ALT glutamic-pyruvic transaminase; AST glutamic oxalacetic transaminase; 25(OH)D 25-hydroxyvitamin D; TG triglyceride; TC total cholesterol; LDL-C low-density lipoprotein cholesterol; HDL-C high-density lipoprotein cholesterol; NAGL neutrophil gelatinase-associated lipocalin; RBP retinol binding protein; RBC red blood cell; ADC apparent diffusion coefficient; ΔADC the cortico-medullary difference in apparent diffusion coefficient; M mesangial hypercellularity; E endothelial hypercellularity; S segmental sclerosis; T interstitial fibrosis/tubular atrophy; C crescent.

Data were presented as the mean ± standard, the median with interquartile range or counts and percentages. A two-tailed *p* < 0.05 was considered statistically significant.

**Figure 2.**
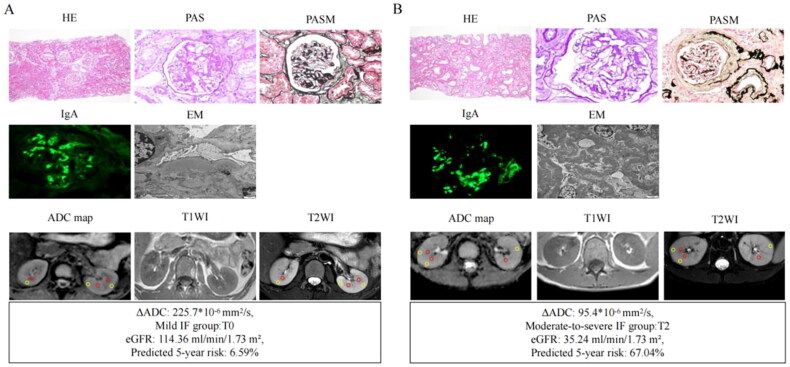
Representative MRI and pathological images of IgAN patients in the high-ΔADC group (A) and low-ΔADC group (B). Two examples of IgAN patients with characteristics (ΔADC value, eGFR, and predicted 5-year risk of kidney progression), diffusion MRI images (ADC, T1WI, and T2WI maps; yellow circles, cortex; red circles, medulla), light microscopy map (HE, PAS, PASM), immunofluorescence map (IgA), and electron microscopy map. HE, hematoxylin-eosin staining; PAS, periodic acid-schiff staining; PASM, periodic acid-silver methenamine staining; EM, electron microscopy; eGFR, estimated glomerular filtration rate.

According to the median value of ΔADC, patients were divided into the high-ΔADC group (>167.1 × 10^−6^ mm^2^/s) and the low-ΔADC group (≤167.1 × 10^−6^ mm^2^/s) (Table 1). Patients in the high-ΔADC group had higher levels of DBP, hemoglobin, 24h urinary calcium while lower levels of medulla ADC compared to the low-ΔADC group (*p* < 0.05). More importantly, the number of patients with predicted 5-year kidney progression (ESKD or a 50% reduction in eGFR) risks > 20% were significantly increased in the low-ΔADC group than high-ΔADC group (23% v.s 7%, *p* = 0.035). The representative DWI images and histopathological images of IgAN patients in the low-ΔADC group and the high-ΔADC group were shown in Figure 2.

### Correlation between ΔADC and clinicopathological parameters

**Figure 3.**
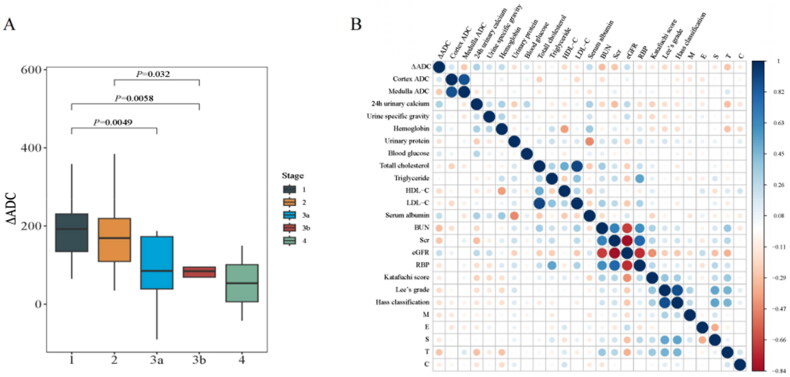
Association of ΔADC and clinicopathological parameters. A. Comparison of ΔADC among IgAN patients at different stages of CKD. B. The correlation between ΔADC and clinicopathological parameters. IgAN, IgA nephropathy; ADC, apparent diffusion coefficient; HDL-C, high-density lipoprotein cholesterol; LDL-C, low-density lipoprotein cholesterol; BUN, blood urea nitrogen; Scr, serum creatine; eGFR, estimated glomerular filtration rate; RBP, retinol binding protein; M, mesangial hypercellularity; E, endothelial hypercellularity; S, segmental sclerosis; T, interstitial fibrosis/tubular atrophy; C, crescent. *<0.05, **<0.01.

As shown in Figure 3A, ΔADC was significantly decreased across the increasing staging of CKD (*p* = 0.0038). More specifically, there was a significant rise in ΔADC at stage G1 relative to later CKD stages (G1 vs G3a: *p* = 0.0049, G1 vs G3b: *p* = 0.0058). Additionally, ΔADC significantly increased at stage G2 relative to stage G3b (*p* = 0.032).

Spearman correlation showed that ΔADC was positively correlated with eGFR (*r* = 0.230, *p* = 0.015), 24h urinary calcium (*r* = 0.310, *p* = 0.001), urine specific gravity (*r* = 0.206, *p* = 0.029), hemoglobin (*r* = 0.247, *p* = 0.009), serum albumin (*r* = 0.247, *p* = 0.009), and negatively correlated with levels of Scr (r=−0.271, *p* = 0.004), BUN (r=−0.304, *p* = 0.001), T score of Oxford classification (r= −0.276, *p* = 0.003) and Lee grades (r=−0.187, *p* = 0.048) (Figure 3B). To further delineate the influence of clinical parameters on ΔADC, univariate and multivariate linear regression analyses were performed on the parameters that significantly correlated with ΔADC. The results revealed that Hemoglobin (β = 0.884, 95% CI = [0.079, 1.690], *p* = 0.032) and eGFR (β = 0.690, 95% CI = [0.137, 1.243], *p* = 0.015) were independent factors of ΔADC values (Table S1).

### Association of ΔADC with eGFR and IF

Further univariate linear regression analyses identified that a number of parameters were significantly associated with eGFR in all enrolled patients (Table 2), including age (β=−0.743, 95% CI = [−1.101, −0.385], *p* < 0.001), SBP (β=−0.319, 95% CI = [−0.618, −0.019], *p* = 0.037), DBP (β=−0.601, 95% CI = [−1.062, −0.14], *p* = 0.011), 24h proteinuria (β=−4.111, 95% CI = [−7.138, −1.084], *p* = 0.008), urinary ammonia (β = 0.411, 95% CI = [0.205, 0.618], *p* < 0.001), 24h urinary sodium (β = 0.089, 95% CI = [0.018, 0.159], *p* = 0.014), 24h urinary calcium (β = 5.044, 95% CI = [2.008, 8.079], *p* = 0.001), RBP (β=−1.106, 95% CI = [−1.296, −0.915], *p* < 0.001), serum cystatin C (β=−21.944, 95% CI = [−28.642, −15.246], *p* < 0.001), and ΔADC (β = 0.101, 95% CI= [0.042, 0.16, *p* = 0.001). When independent variables were selected by a backward stepwise multivariate linear regression method with a *P*-value threshold of 0.05, ΔADC was still independently associated with eGFR (β = 0.04, 95% CI = [0.003, 0.077], *p* = 0.033) (Table 2).

**Table 2.** Univariate and multivariate linear regression analysis for eGFR in IgAN patients.

Parameter	Univariate		Multivariate^a^	
	β(95%CI)	*P* value	β(95%CI)	*P* value
Age	−0.743 (−1.101, −0.385)	<0.001	−0.627 (−0.863, −0.392)	<0.001
Gender	−4.982 (−15.422,5.457)	0.346		
BMI	−0.172 (−1.269,0.925)	0.757		
SBP	−0.319 (−0.618, −0.019)	0.037		
DBP	−0.601 (−1.062, −0.14)	0.011	−0.257 (−0.525,0.012)	0.061
Serum albumin	0.668 (−0.286,1.621)	0.168		
Hemoglobin	0.195 (−0.079,0.47)	0.161		
24h proteinuria	−4.111 (−7.138, −1.084)	0.008	−1.472 (−3.416,0.473)	0.136
Urinary ammonia	0.411 (0.205,0.618)	<0.001	0.114 (−0.034,0.262)	0.129
24h Urinary Sodium	0.089 (0.018,0.159)	0.014	0.042 (0,0.084)	0.042
24h Urinary Calcium	5.044 (2.008,8.079)	0.001	2.309 (0.256,4.361)	0.028
24h Urinary Magnesium	1.11 (−2.434,4.653)	0.536		
24h Urinary Phosphorus	0.213 (−0.442,0.868)	0.52		
RBP	−1.106 (−1.296, −0.915)	<0.001	−0.831 (−1.019,−0.644)	<0.001
25(OH)D	0.015 (−0.285,0.314)	0.924		
ΔADC (× 10^-6^ mm^2^/s)	0.101 (0.042,0.16)	0.001	0.04 (0.003,0.077)	0.033

eGFR estimated glomerular filtration rate; BMI body mass index; SBP systolic blood pressure; DBP diastolic blood pressure; RBP retinol binding protein; 25(OH)D 25-hydroxyvitamin D; ADC apparent diffusion coefficient; ΔADC the cortico-medullary difference in apparent diffusion coefficient.

^a^ Multivariable: adjusted for age, DBP, 24h proteinuria, Urinary ammonia, 24h Urinary Sodium, 24h Urinary Calcium, RBP and Delta ADC.

**Figure 4.**
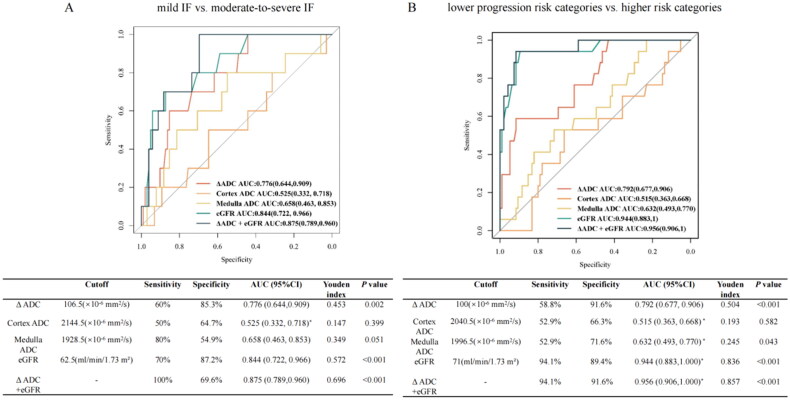
ROC curves for the diagnostic performance of DWI parameters for discriminating IF severity and 5-year progression risks. Diagnostic value of different DWI parameters for differentiating T1-2 from T0 (A) and differentiating patients with the predicted 5-year progression risks ≤ 20% from > 20% (B). *, significant (*p* < 0.05) v.s ΔADC using pairwise comparison of ROC curve analysis. ADC, apparent diffusion coefficient; IF, interstitial fibrosis; AUC, the area under the ROC curve; DWI, diffusion weighted imaging; eGFR, estimated glomerular filtration rate.

Besides, to illustrate the association of ΔADC with kidney fibrosis, receiver-operating characteristic (ROC) analysis was performed. ΔADC showed an AUC of 0.776 for discriminating T1-2 from T0, with a sensitivity of 60% and a specificity of 85.3% (Figure 4A). The optimal cutoff value of ΔADC for predicting moderate-to-severe IF was 106.5 × 10^−6^ mm^2^/s. In addition, cortex ADC and medulla ADC had no significant ability in discriminating IF severity (*p* > 0.05). Meanwhile, a pairwise comparison of ROC curves showed that ΔADC and eGFR had similar performance in differentiating T1-2 v.s T0 (eGFR: AUC = 0.884, *p* > 0.05 vs. ΔADC). Moreover, the combination of ΔADC and eGFR displayed an AUC of 0.875 with 100% sensitivity and 69.6% specificity to well discriminate moderate-to-severe IF from mild IF.

### Predictive value of ΔADC on 5-year kidney progression risk

As shown in Figure 4B, ROC analysis was conducted to confirm the predictive value of ΔADC in the prognostic risk stratification. ΔADC showed an AUC of 0.792, indicating good accuracy in distinguishing between higher and lower progression risk categories, with a high specificity (91.6%) but a moderate sensitivity (58.8%). Based on a risk-prediction tool, the optimal cutoff value of ΔADC for predicting higher 5-year kidney progression risk was 100 × 10^−6^ mm^2^/s, as calculated by obtaining the best Youden index. According to the pair wise ROC analysis, ΔADC showed better performance compared to cortex or medulla ADC in discriminating higher v.s lower progression risk categories (*p* < 0.05). Adding ΔADC to eGFR showed the largest AUC of 0.956 (sensitivity = 94.1%, specificity = 91.6%) for stratifying prognostic kidney progression risk, followed by eGFR (AUC = 0.944, sensitivity = 94.1%, specificity = 89.6%). The comparison of clinicopathological features according to the optimal cutoff value of ΔADC (100 × 10^−6^ mm^2^/s) were shown in Supplementary Table 2.

Consistently, when stratified patients by median values of ΔADC, low-ΔADC group (≤ median value 167.1 × 10^−6^ mm^2^/s) had 3.930-times risk for higher progression risk categories (95% CI 1.285-14.755, *p* = 0.024) compared with the high-ΔADC group (> median value 167.1 × 10^−6^ mm^2^/s) in unadjusted models. After adjusting for age, gender, and duration of kidney disease, the association was still significant with an OR of 3.417 (95% CI 1.088-13.062, *p* = 0.047, model 2 in Table 3). And this increased risk remained in low-ΔADC, even after extensive adjustment for model 2 plus MAP, 24h urinary protein, use of RAASi, and use of immunosuppressive therapy (OR 7.509, 95% CI 1.585-48.460, *p* = 0.018, model 3 in Table 3). Consistently, additional reclassification analyses revealed that after adding age, gender, and duration of kidney disease to ΔADC (model 2), it resulted in a 19.9% improvement (95% CI 0.50-44.50) in NRI compared with model 1 in Table 3. Moreover, the addition of important clinical and demographic factors (model 3 in Table 3) could greatly improve the model performance, with a 74.9% (95% CI 51.4-94.8) compared with model 1 and a 72.7% (95% CI 42.7-99.4) improvement compared with model 2 in Table 3, respectively.

**Table 3.** Prediction performance of ΔADC for 5-year kidney progression risk.

Variable	ΔADC (× 10^−6^ mm^2^/s)		*P* value	NRI, % (95%CI)	
	≤ 167.1 (*n* = 56)	>167.1 (*n* = 56)			
Number of events, %	13(23)	4(7.1)	0.035	–	–
Model 1 OR (95%CI)	3.930 (1.285, 14.755)	Reference	0.024	Reference	–
Model 2 OR (95%CI)	3.417 (1.088, 13.062)	Reference	0.047	19.9 (0.5, 44.5)	Reference
Model 3 OR (95%CI)	7.509 (1.585, 48.460)	Reference	0.018	74.9(51.4, 94.8)	72.7 (42.7, 99.4)

Odds ratios (OR) and 95% confidence intervals were derived from Multinomial logistics regression models.

Model 1: unadjusted.

Model 2: adjusted for age, gender, and duration of kidney disease.

Model 3: Model 2 plus MAP, 24h urinary protein, use of RAASi, and use of immunosuppressive therapy.

MAP = (systolic blood pressure + 2* diastolic blood pressure)/3.

ΔADC the cortico-medullary difference in apparent diffusion coefficient; MAP, mean arterial pressure; RAASi, renin-angiotensin-aldosterone system inhibitors; AUC area under the curve of ROC; NRI net reclassification improvement. A two-tailed *p* < 0.05 was considered statistically significant.

## Discussion

Our findings supply evidence for the role of ΔADC in assessing the clinical, pathological, and prognostic risk stratification of IgAN patients. First, in 112 biopsy-confirmed IgAN patients, ΔADC significantly decreased with higher CKD stages. It was significantly correlated with parameters of renal function, Lee grades, and kidney IF. Second, ΔADC was independently associated with eGFR by backward stepwise multivariate linear regression analyses. Thirdly, ΔADC showed the ability in evaluating kidney IF severity but the reliability needs to be validated. And the combination of eGFR and ΔADC enhanced diagnostic sensitivity, reaching 100%. More importantly, after adjusting confounding factors, lower levels of ΔADC were significantly associated with increased risk of higher predicted 5-year kidney progression risk (>20%), suggesting that ΔADC was an independent determinant for kidney progression in IgAN. Meanwhile, ΔADC identified patients with higher 5-year kidney progression risk estimates with satisfactory accuracy. Our findings were robust that reclassification analysis confirmed ΔADC’s strong prognostic value for stratifying future kidney progression risk in IgAN patients.

To date, accumulating data have pointed to the important role of diffusion MRI parameters in kidney IF evaluation. Using the Oxford MEST-C classification to score the IF in CKD patients, Mao W et al. established that ADC values had an AUC of 0.87 and 0.93 respectively for the discrimination of ≤25% vs. > 25% and ≤50% vs. > 50% IF [26]. In another cohort of 46 biopsy-proven IgAN patients, cortex and medulla ADC showed an AUC of 0.877 and 0.854, respectively, for differentiating Oxford T0 from T1/T2, and both had a significant correlation with T scores [27]. Also, several other studies have consistently reported a negative correlation between ADC and renal fibrosis assessed by biopsy in CKD or kidney allografts [10, 24, 25, 28, 29]. Furthermore, in recent decades, great progress has been made in evaluating kidney fibrosis by cortico-medullary ADC difference (ΔADC). ΔADC, introduced by Friedli I et al. has proved several advantages to minimizing the physiological inter-individual variation and optimizing kidney IF assessment [17, 30]. Moreover, growing research demonstrated that ΔADC was reproducible and correlated better to IF assessed by standard histology than absolute cortical or medullary ADC values in CKD and transplant patients [17–19]. Consistent with these findings, the current study further confirmed the association between ΔADC and the assessment of kidney IF in IgAN patients with several different methods. Using Spearman correlation analyses, ΔADC was proved to be significantly correlated with T scores. By ROC analyses, ΔADC showed a reliable ability to evaluate IF severity. And the diagnostic performance to discriminate IF was improved by adding eGFR to ΔADC with a perfect sensitivity (100%) which suggested kidney imaging would be an additional method to eGFR estimation for the detection of early kidney lesions. When dividing patients into two groups according to the optimal cutoff value of ΔADC for 5-year kidney progression risk, patients in the low-ΔADC group had more severe IF than the high-ΔADC group. Furthermore, previous studies have validated that Oxford T score highly predicts renal prognosis in IgAN patients [31, 32]. And T score was suggested as the only variable associated with ESKD in the Oxford classification [33]. Taken together, our results supplied solid evidence for the role of ΔADC as a noninvasive and well-applied tool to predict and evaluate kidney fibrosis, helping to tailor treatment and judge renal prognosis in IgAN.

Another important finding in the current study was that ΔADC was independently associated with eGFR, the most commonly used biomarker to evaluate renal function in clinical practice in IgAN patients. Consistent with our results, Berchtold L et al. found that ΔADC was highly correlated to eGFR in CKD and allograft patients [19]. Besides, several studies also proved the association between ADC values and eGFR. One prospective, case-control study suggested that whole kidney ADC correlated with eGFR in CKD patients [9]. Another exploratory study also indicated that baseline cortical ADC was associated with the change in eGFR over time, and the MRI parameter remained stable over 1 year in CKD patients [34]. Moreover, diffusion MRI did not require the contrast medium and could provide quantitative parameters to assess the change of renal microstructure and function in the all-staging CKD population [19]. Therefore, ΔADC could provide additional information on eGFR estimation and be a trustworthy noninvasive indicator reflecting kidney function.

Moreover, ΔADC showed reliable predictive ability for stratifying 5-year kidney progression risk (≤ v.*s* > 20%). The predicted risk of developing ESKD or decreasing of eGFR >50% within 5 years was calculated in accordance with the formula recommended by the KDIGO 2021 Clinical Practice Guideline [8]. Clinical and pathological factors readily available in clinical practice, including age, race, eGFR, SBP and DBP, proteinuria, use of RAASi and immunosuppression therapy at the time of biopsy, MEST-C score of Oxford classification were considered in the risk-prediction tool [7, 8]. Based on the well-applied tool in IgAN, our results indicated that low ΔADC was associated with increased odds of predicted 5-year progression risk >20% independent of important clinicopathological indicators. With the high specificity and moderate sensitivity of cutoff value, ΔADC would help to boost the discriminative strength as a potential tool for identifying cases who may have higher predicted probabilities of future kidney development. The finding was supported by a previous study that ΔADC outperformed ADC values for the prediction of renal function evolution and was a predictor of kidney function decline and dialysis initiation in CKD patients due to the ability to cumulate the effects of capillary rarefaction, perfusion, and fibrosis involving in CKD development [11]. Another study also demonstrated that cortex and medulla ADC values had an AUC of 0.807 and 0.780, respectively, differentiating the 5-year risk (≤10% v.*s* > 10%) in IgAN patients [27]. Consistently, our study found that ΔADC displayed better prediction ability in CKD progression compared to the cortex or medulla ADC. And cortex and medulla ADC values showed an AUC of 0.515 and 0.632, respectively, differentiating the 5-year risk (≤20% v.*s* > 20%) in our patients. The CKD stages of patients and sample size may explain the difference as there were 32.60% of patients with eGFR < 60 in Liang’s study (*n* = 46) versus 13.39% in our study (*n* = 112) [10]. Furthermore, our model that adding ΔADC to eGFR could identify patients with higher 5-year kidney progression risk (>20%) with both excellent sensitivity (91.6%) and specificity (94.1%). Hence, ΔADC is a valuable, noninvasive complementary tool for identifying IgAN patients at high risk of kidney disease progression, particularly for those unable to undergo biopsy. Based on ΔADC adding to important clinicopathological indicators, we could easily divide IgAN patients into different risk categories, guiding the optimized clinical decision-making and individualized prevention.

Our study must be interpreted in light of several limitations. First, it was a monocentric study design, and the number of enrolled IgAN patients was relatively small, which was insufficient to validate the model in independent data sets. Second, the enrolled patients presented with preserved renal function and mild proteinuria in which it may take a long follow-up period to observe the kidney endpoints in the real world. So the prognostic risks for kidney disease progression were calculated by the risk-prediction tool of IgAN in the current study. Future work would focus on continuing follow-up and exploring the association between ΔADC and actual kidney outcomes. Third, only the role of ΔADC in the assessment of IgAN was evaluated in our study. Comparison of multiple kidney functional MRI parameters may supply more information on risk stratification and the clinical decision-making of IgAN.

Overall, we found that ΔADC was significantly associated with eGFR and enabled a reliable evaluation of kidney IF severity in IgAN patients. Low ΔADC was predictive of high 5-year kidney progression risk, independently of important clinical factors including age, gender, duration of kidney disease, MAP, 24h urinary protein, use of RAASi and use of immunosuppressive therapy. Moreover, the predictive ability to identify patients at high risk of severe kidney fibrosis and adverse progression is estimated with satisfactory accuracy, making ΔADC a non - invasive tool for complementarily evaluating disease activity and the prognostic risk stratification in patients with IgAN. However, its practicality needs further validation.

## Supplementary Material

Supplementary Table241206.docx

## Data Availability

The datasets used and analyzed during the current study are available from the corresponding author upon reasonable request.
