# Supplementary material for: Delta corticomedullary apparent diffusion coefficient on MRI as a biomarker for prognosis in IgA nephropathy
Source: Ren Fail. 2024 Dec 17;47(1):2441394. doi: 10.1080/0886022X.2024.2441394 (PMC11654037; doi:10.1080/0886022X.2024.2441394)
Supplement: Supplementary Table241206.docx [file IRNF_A_2441394_SM9847.docx]

Supplementary Table 1. Univariate and Multivariate linear regression analysis for ΔADC in IgAN patients

| \| Parameter \| \| --- \| \|  \| | Univariate | | Multivariate ^a^ | |
| --- | --- | --- | --- | --- | --- | --- |
|  | β（95%CI） | *P* value | β（95%CI） | *P* value |
| Hemoglobin | 1.119(0.306,1.931) | 0.007 | 0.884(0.079,1.69) | **0.032** |
| Serum albumin | 3.901(1.057,6.745) | 0.008 | 2.593(-0.196,5.383) | 0.068 |
| BUN | -13.615(-20.63,-6.6) | < 0.001 |  |  |
| Scr | -0.78(-1.178,-0.382) | < 0.001 |  |  |
| eGFR | 0.945(0.396,1.494) | 0.001 | 0.69(0.137,1.243) | **0.015** |
| Urine specific gravity | 2807.655(160.499,5454.811) | 0.038 |  |  |
| 24h Urinary Calcium | 12.81(3.426,22.195) | 0.008 |  |  |

BUN blood urea nitrogen; Scr serum creatine; eGFR estimated glomerular filtration rate.

^a^ Multivariable: adjusted for Hemoglobin, Serum albumin, eGFR, Urine specific gravity, 24h Urinary Calcium.

Supplementary Table 2. Clinical and radiological characteristics according to the cutoff values of ΔADC.

| Parameter | ΔADC≤ 100×10^-6^ mm^2^/s  (n=18) | ΔADC >100×10^-6^ mm^2^/s  (n=94) | *P* value |
| --- | --- | --- | --- |
| Age (years) | 44.22 ± 15.51 | 39.24 ± 13.25 | 0.216 |
| Gender(male/female) | 11/7 | 44/50 | 0.393 |
| Duration of kidney disease (years) | 4 (2.25, 12) | 8.5 (1, 24) | 0.981 |
| **CKD stage**  **(1/2/3a/3b/4)** | 5/4/4/4/1 | 67/21/4/1/1 | **< 0.001** |
| **Clinical parameter** |  |  |  |
| Body mass index (kg/m2) | 20.45 (17.4, 25.12) | 21.1 (18.2, 25.4) | 0.806 |
| SBP (mmHg) | 135 (122, 150) | 135.5 (121, 147) | 0.994 |
| DBP (mmHg) | 85.5 (77.75, 102) | 92 (83.25, 102) | 0.421 |
| **Laboratory parameter** |  |  |  |
| Urinary protein (g/d) | 1.35 (0.3, 2.9) | 0.6 (0.3, 1) | 0.081 |
| eGFR (ml/min/1.73 m²) | 56.5 (38.75, 92) | 102 (83, 113.75) | **< 0.001** |
| BUN (mmol/L) | 7.24 (5.82, 9.01) | 4.82 (4.2, 5.78) | **< 0.001** |
| Scr (μmol/L) | 121.5 (80.75, 139.25) | 76 (58.25, 93) | **< 0.001** |
| Hemoglobin (g/L) | 125.61±23.25 | 134.36±17.9 | 0.145 |
| AST (u/L) | 22 (18.95, 26.75) | 19.15 (17.12, 22.32) | **0.018** |
| ALT (u/L) | 16.7 (12.25, 20.42) | 14.4 (11.03, 20.2) | 0.539 |
| Serum albumin (g/L) | 35.5 (32, 37.75) | 38 (36, 41) | **0.013** |
| Fasting blood glucose (mmol/L) | 4.27±0.59 | 4.52±0.6 | 0.112 |
| Triglyceride (mmol/L) | 1.19 (0.97, 1.67) | 1.2 (0.93, 1.76) | 0.997 |
| Total cholesterol (mmol/L) | 5.15 (4.21, 5.61) | 4.56 (3.95, 5.17) | 0.149 |
| LDL-C (mmol/L) | 3.16 (2.54, 3.62) | 2.87 (2.41, 3.24) | 0.328 |
| HDL-C (mmol/L) | 1.2 (1.05, 1.37) | 1.06 (0.92, 1.3) | 0.083 |
| Urine titratable acid | 15.5 (12, 30.25) | 18 (12, 28.75) | 0.521 |
| Urine ammonia | 20.5 (16.25, 37.25) | 36.5 (26, 56) | **0.015** |
| 25(OH)D (nmol/L) | 33 (23, 37.5) | 34.5 (26, 42.75) | 0.286 |
| Serum NAGL (ng/mL) | 135.25 (87.83, 222.1) | 77.25 (54.5, 146.7) | **0.04** |
| IgG (g/L) | 9.88±2.92 | 10.83±2.67 | 0.21 |
| IgA (g/L) | 2.69 (2.31, 3.53) | 3 (2.39, 3.59) | 0.566 |
| IgM (g/L) | 1.03 (0.78, 1.45) | 1.08 (0.81, 1.42) | 0.868 |
| C3 (g/L) | 0.78 (0.73, 0.91) | 0.84 (0.77, 0.98) | 0.187 |
| C4 (g/L) | 0.24 (0.2, 0.28) | 0.22 (0.18, 0.26) | 0.458 |
| RBP (mg/L) | 57.6 (40.65, 75.18) | 40.7 (32.3, 48.45) | **0.014** |
| Urinary RBC (10 thousand/ml) | 6 (3.6, 11.28) | 4.45 (1.55, 16.95) | 0.902 |
| 24h Urinary Calcium | 1.51 (0.95, 2.03) | 2.64 (1.61, 3.93) | **0.003** |
| 24h Urinary Magnesium | 3.09 (2.19, 3.76) | 3.1 (2.26, 3.88) | 0.843 |
| 24h Urinary Phosphorus | 13.76 (9.05, 18.75) | 15.7 (11.72, 20.7) | 0.326 |
| 24h Urinary Sodium | 106.45 (84.42, 126.33) | 116.65 (86.62, 161.9) | 0.421 |
| 24h Urinary Chlorine | 106.15 (60.85, 122.83) | 112.05 (88.22, 149.25) | 0.178 |
| 24h Urinary Potassium | 31.15±17.26 | 30.1±9.83 | 0.805 |
| Cortex ADC (× 10^-6^ mm^2^/s) | 2001 (1884, 2148.75) | 2106.5 (2007.25, 2208) | 0.073 |
| Medulla ADC (× 10^-6^ mm^2^/s) | 1976.5 (1858.5, 2088.5) | 1923.5 (1836.5, 2021) | 0.235 |
| ΔADC (× 10^-6^ mm^2^/s) | 44.89 ± 54.03 | 192.64 ± 67.56 | **< 0.001** |
| **Pathological feature** |  | ` |  |
| Katafuchi score  (0/1/2/3/4) | 5/1/3/6/3 | 35/11/27/16/5 | 0.18 |
| Lee’s grade (II/III/IV) | 1/15/2 | 9/89/3 | 0.207 |
| Haas classification  (I/II/III/IV) | 1/15/0/2 | 9/81/1/3 | 0.358 |
| Oxford classification |  |  |  |
| M (0/1) | 5/13 | 36/58 | 0.561 |
| E (0/1) | 17/1 | 89/5 | 1 |
| S (0/1) | 0/18 | 9/85 | 0.35 |
| T (0/1/2) | 14/2/2 | 88/6/0 | **0.014** |
| C (0/1/2) | 15/2/1 | 84/9/1 | 0.259 |
| **Predicted 5-year risk** |  |  |  |
| >5%, n (%) | 15 (83) | 59 (63) | 0.157 |
| >10%, n (%) | 12 (67) | 22 (23) | **< 0.001** |
| >20%, n (%) | 10 (56) | 7 (7) | **< 0.001** |

CKD chronic kidney disease; BMI body mass index; SBP systolic blood pressure; DBP diastolic blood pressure; eGFR estimated glomerular filtration rate; BUN blood urea nitrogen; Scr serum creatine; ALT glutamic-pyruvic transaminase; AST glutamic oxalacetic transaminase; 25(OH)D 25-hydroxyvitamin D; TG triglyceride; TC total cholesterol; LDL-C low-density lipoprotein cholesterol; HDL-C high-density lipoprotein cholesterol; NAGL neutrophil gelatinase-associated lipocalin; RBP retinol binding protein; RBC red blood cell; ADC apparent diffusion coefﬁcient; ΔADC the cortico-medullary difference in apparent diffusion coefficient; M mesangial hypercellularity; E endothelial hypercellularity; S segmental sclerosis; T interstitial ﬁbrosis/tubular atrophy; C crescent.

Data were presented as the mean±standard, the median with interquartile range or counts and percentages. A two-

tailed *P*<0.05 was considered statistically significant.
